# Editorial Department

**Published:** 1866-05

**Authors:** 


### Editorial Department.

Principles and Practice of Medicine. By Austin Flint, M. D., Professor of the Principles and Practice of Medicine in the Bellevue Hospital Medical College and in the Long Island College Hospital; Fellow of the New York Academy of Medicine, etc., etc.

We have been awaiting the appearance of this work with great expectation that it would furnish the practitioner a correct therapeutical guide, which desideratum has not heretofore been furnished the profession. The practice of medicine has so wholly changed within the last twenty years, that the older authors although correct in their descriptions of disease and in the pathological conditions represented, are yet recommending a plan of treatment which recent and more carefully conducted observation indicates to be not only unnecessary but often injurious. Great changes have taken place in our views and much progress made in our knowledge of the nature and causes of disease; its natural tendency to recovery has hardly been discovered until recently, or if understood it has not been acted upon in therapeutics; even now it is only half appreciated by the most enlightened and progressive, while the less observing continue to treat disease as a unity and with mistaken earnestness "strike both it and nature at the same time," or with astounding credulity prescribe inert remedies and attribute to their influence results which belong wholly to other causes.

Dr. Flint has attempted no new plan, but has followed the usual course in presenting his work, which embraces the subjects usually taught in the lecture room. He has an original and unexcelled style of description and presents his subjects in so clear and elegant a manner as to both entertain and instruct at the same time.

We had proposed to follow our author in his special therapeutics and tell our readers what he says about the treatment of disease, but our space will not at present admit of such notice. He has not recommended blood letting, calomel, ipecac and antimony, for all inflammatory disease, but has modestly intimated that it is better practice to take some good care of our patients at the same time that treat their diseases. He has shown defference for the opinions of others and treated those who differ from him with great consideration; in speaking of remedies he has often given a place and spoken hardly, disapprovingly, of medicines which he yet betrays no sympathy for, in themselves considered, which are admitted—recognized on account of their friends. He says, "A great change has taken place, within the last few years, with respect to blood-letting in the treatment of acute inflammations. This measure was formerly thought to be highly important, and was rarely omitted. It is now considered by many as seldom, if ever, called for. The infrequent use of the lancet now, contrasted with its frequent use twenty-five years ago, constitutes one of the most striking of the changes in the practice of medicine, which have occurred during this period. It can hardly be doubted that this measure was formerly adopted too indiscriminately, and often employed too largely; but, with the natural tendency to pass from one extreme to another, it may be that the utility of blood-letting in certain cases, at the present time, is not sufficiently appreciated.

"Experience and pathological reasoning combine to show that blood-letting does not exert a direct controlling effect upon an inflammatory disease. It may exert a powerful immediate effect as a palliative measure, and whatever curative power it may possess is exerted indirectly. Its therapeutic action consists in lessening the frequency and force of the heart's action; in other words, in diminishing the intensity of symptomatic fever. In the early period of an acute inflammation accompanied by high febrile movement, as indicated by a pulse accelerated and of abnormal strength, the abstraction of blood affords relief, and may contribute to a favorable progress of the disease. It should enter into the treatment of a certain proportion of cases, provided other and more conservative means for the same ends are not available.

"The evils of blood-letting arise from its spoliative effect upon the blood. It diminishes the red corpuscles, and these, during the progress of an acute disease, are not readily reproduced. It induces, thus, the anæmic condition, and in this way impairs the vital powers. It will be likely to do harm, therefore, whenever it is important to economize the powers of life, and it may contribute to a fatal result in diseases, or cases of disease, which involve danger of death by asthenia.

"The opinion is held by some that diseases, and the human constitution, have undergone a notable change during the last quarter of a century, and that blood-letting and other antiphlogistic measures are less appropriate now than formerly, on this account. This opinion seems to me not well founded. After a professional experience extending beyond this period just named, I do not hesitate to express a conviction that acute inflammations at the present day are essentially the same as they were twenty-five years ago, and that antiphlogistic measures were no more appropriate then, than now.

"Were it true that such changes have occurred, the fact would strike at the root of medical experience. If changes requiring a revolution in therapeutics are liable to occur with each successive generation, it is evident there can be no such thing as permanent principles of practice in medicine; the fruits of experience in our day, which so many are striving to develop, will be of no utility to those who are to come after us.

"The other measures belonging to the antiphlogistical denomination are treated, as we have said, considerately; but very few conditions are described which may not be more appropriately treated with milder and less objectionable remedies."

This work is sufficiently comprehensive to include a consideration of all the usual topics included in works upon the practice of medicine, with descriptions of disease, which are plain and truthful and according to the most recent and scientific views.

The great practical experience of the author, his careful observation of the phenomena of disease, and his habit of accurately noting its varying manifestations during a long period of professional life, together with his distinguished abilities as a teacher and writer, combine to make this book of inestimable value, as the recorded experience of one of the clearest and best educated minds ever devoted to the theory and practice of medicine. Dr. Flint's *Theory and Practice of Medicine* will be eagerly perused by all our readers—will be regarded as the *Bible* of practical medicine.

---

Rhinoscopy aud Laryngoscopy: Their Value in Practical Medicine. By Dr. Friederich Shmelder, Physician in Ordinary to His Majesty the Emperor of Mexico, Member of the Royal Medical Society of Vienna, and of the Medical Society of the Pantheon of Paris, formerly Member of the Medical Faculty of the University of Vienna, and Surgeon to the Branch Hospital and Guinpenderf. Translated from the German by Edward Caswell, M. D. With wood-cuts and two chromo-lithographic plates. New York: William Wood & Co., 61 Walker street, 1866.

This work is placed before the medical profession so as to stimulate it to become better acquainted with the science of which it treats and the use of the instruments it describes. The author makes no undue pretensions, and shows a thorough familiarity with the subjects he discusses. The pathology of the larynx has gained a rich prize from the practical application of the laryngoscope; "and this has partly been made possible only by the fact that we could correct our previous ideas upon many of the physiological relations of the air-passages and of the parts surrounding its commencement. The therapeutics of the diseases of the larynx is, so to speak, newly created; on the one hand, we have gained new and established indications, on the other hand, local treatment has passed out of the condition of deceptive groping upon that of manipulation, which can be counted upon and watched over. New types of disease have been discovered, now that we are in a position to perceive alterations, no trace of which can be found upon the cadaver, or in which only the very last stages of development there meet us."

According to the author's method, the apparatus for making a laryngial examination is exceedingly simple, and consists of the laryngial mirrors, tongue-spatulas and illuminating spectacles. Large mirrors are preferable, as they give a clearer and more perfect image, but if from consideration of space, the smallest mirrors become necessary, the author prefers the metalic mirrors, as nothing of the reflecting surface is lost by a narrow setting. In the introduction of the laryngial mirror, the author gives the following rules: "The uvulæ should rest upon the back of the mirror, and with the soft palate is pressed backwards and upwards; the lower edge of the mirror is then gently pressed back upon the posterior wall of the pharynx, while its stem lies in the angle of the mouth, behind the superior canine teeth. The mirror should stand symmetrically and look exactly downwards and forwards. Every mirror should be warmed before its introduction. The image of the larynx in the mirror shows the right vocal chord of the person examined at the left of the observer. In the same manner all these parts which in the object stand in front, as the tongue, epiglottis, the anterior extremity of the vocal chords, the anterior wall of the trachea are seen in the mirror above, and at the same time somewhat forward, while, on the other hand, all parts which in the object lie behind, are seen below and behind in the mirror, as the arytenoid cartilages, the posterior extremities of the vocal chords, and the commencement of the œsophagus." It has been the author's experience that in about five out of a hundred individuals a laryngoscopic examination cannot be made, and that in only twenty-five per cent. does the examination succeed the first time. This work will prove very instructive to all readers, especially so to those pursuing this branch of practice.

---

The Physiology of Man; designed to represent the Existing State of Physiological Science, as applied to the Functions of the Human Body. By Austin Flint, jr., M. D., Professor of Physiology and Microscopy in the Bellevue Hospital Medical College, New York, and in the Long Island College Hospital; Fellow of the New York Academy of Medicine, Microscopist to the Bellevue Hospital. New York: D. Appleton & Co., 1866.

The plan of this work will comprise four volumes, based on the natural sub-division of the subject, the present volume embracing an introduction—the blood, circulation and respiration.

The treatise on the Proximate Principals in the introduction is very elaborate, and presents many new facts. The division of them is that adopted by Robin and Verdeil, with but slight modification into first, Inorganic Substances; secondly, Organic Non-Nitrogenized Substances; and thirdly, Organic Nitrogenized Substances. In summing up a review of the functions of the inorganic constituents of the body, excluding the gases, the author divides them into two groups. "One which is composed of those substances existing particularly in the solids and semi-solids, which are in a condition of molecular union with organic substances, merge their identity, as it were, into them, and become necessary constituents of the tissues; and the other, composed of substances which rather regulate by their influence in endosmosis, or otherwise the nutritive processes do not seem to be indispensable constituents of the tissues, but have rather an accessory office to perform in the functions of nutrition." At the head of the first group he places water, while at the head of the second group stands chloride of sodium. The substances entering into the formation of the first group form a considerable proportion of the body, and are discharged from it in small quantities, while of those of the second group but little is retained in the organism, the largest proportion being thrown off.

Under the second classification a number of reliable and readily applicable tests for detecting the presence of sugar in the animal tissues are described, which to a practical physician are useful, as there are many pathological conditions in which it is essential to ascertain the facts of its presence or non-presence in the body.

In the quantitative analysis of the organic constituents of the blood a new method has been presented for which the author claims the advantage over other analysis of simplicity and facility of application. Repeated experiments have, with but slight differences, verified his estimates, and "that the extreme accuracy desired by chemists is not desirable for the physiologist, as even an approximation of the proportions of the organic matter, as they really exist, is better than the most accurate estimate of their dry residue." Anatomically he considers the plasma and the red corpuscles the most important elements of the blood; the former seeming to be employed in the nourishment of the tissues, while the latter seem to be intimately connected with the functions of respiration, their chief office being to carry the oxygen from the lungs to the tissues. Chemically the plasma contains the elements required for the regeneration of all the parts of the body. These elements are continually used up in nutrition, and replaced by the absorption of articles of food after they have undergone the process of digestion.

The changes which the blood undergoes in respiration has received much attention and investigation from the writer. Especially does the analysis of the blood for gases receive attention. He says, "during fasting the arterial blood contains from nine to eleven parts per hundred of oxygen, which amount is raised from seventeen to eighteen, and even twenty parts per hundred during digestion. The quantity of carbonic acid is even more variable than that of oxygen. During digestion the arterial blood contains from five to six parts per hundred of carbonic acid, of which amount after fasting for twenty-four hours frequently "*not a trace can be discovered.*" The nitrogen of the blood does not appear to perform any important office in the process of respiration. Its proportion is larger in the arteries than in the venous blood.

In conclusion we recommend to the student and the practitioner of medicine this work on a science which must be conceded to be the basis of true pathology. It is full cf well established, well selected facts and principles; contains what is known in physiological science and is written in the excellent style of the eminent teacher and discoverer of physiological truth.

